# Mediterranean diet, eating behaviours, and nutritional intervention in childhood obesity: what works, where, and for whom–a narrative review

**DOI:** 10.3389/fnut.2026.1892494

**Published:** 2026-09-21

**Authors:** Julia Bober, Ewelina Gaszyńska

**Affiliations:** 1 International Doctoral School, Medical University of Lodz, Lodz, Poland; 2 Department of Nutrition and Epidemiology, Medical University of Lodz, Lodz, Poland

**Keywords:** CEBQ, childhood obesity, KIDMED, lifestyle intervention, Mediterranean diet

## Abstract

Childhood obesity is a leading public health challenge with rising global prevalence and significant impacts on cardiometabolic health extending into adulthood. Lifestyle intervention, with nutrition as its central component, is the main point of paediatric obesity management, yet the evidence base remains fragmented across regions, instruments, and clinical models. This narrative review synthesises European evidence on Mediterranean diet (MD) adherence assessment, multidisciplinary intervention outcomes, and global comparative patterns in paediatric obesity management. Among dietary patterns, the Mediterranean diet (MD) remains the most evidence-supported framework for cardiometabolic prevention in children and adolescents. KIDMED, a 16-item validated instrument, has been adapted for multiple European paediatric populations and consistently identifies low legume, whole grain, and extra-virgin olive oil intake as the principal adherence gaps associated with a higher body mass index (BMI) Z-score, as well as a higher age- and sex-specific BMI percentile. The Children’s Eating Behaviour Questionnaire (CEBQ) provides a complementary behavioural marker with food-approach subscales positively associated with BMI Z-score and food-avoidance subscales inversely associated, a pattern replicated across European, Latin American, and Middle Eastern cohorts. Structured multidisciplinary interventions consistently produce modest reductions in BMI Z-score, typically between 0.10 and 0.25 units over 3 to 12 months. The strongest predictors of response are younger age, lower baseline obesity severity, breakfast adherence, and the absence of obese parents. Low KIDMED score additionally functions as a screening signal for paediatric Metabolic Dysfunction-Associated Fatty Liver Disease (MAFLD). Continental comparisons suggest that dietary-quality predictors of paediatric obesity are broadly consistent across regions. However, the feasibility of Mediterranean-style approaches varies substantially with local food availability, culinary traditions and economic access, so cross-cultural application of KIDMED-based screening requires careful local adaptation. The review concludes that routine integration of KIDMED and CEBQ within structured multidisciplinary pathways represents an evidence-informed, implementation-ready step for pediatric obesity care, particularly in under-studied Central and Eastern Europe.

## 1 Introduction

Childhood obesity is a non-communicable disease of multifactorial origin, defined by the World Health Organization (WHO) as excess body weight that presents a risk to health and implemented in children and adolescents through age- and sex-specific body mass index (BMI) Z-score cut-offs (1, 2). Eating behaviours, dietary patterns and metabolic trajectories established during childhood and adolescence shape lifelong cardiometabolic risk and predict the development of cardiovascular disease, metabolic syndrome, type 2 diabetes and other lifestyle-related conditions in adulthood (3, 4). The literature to date has focused predominantly on adult dietary intervention. The paediatric population, and specifically children with obesity in Central and Eastern Europe, remains under-studied (5). This under-representation concerns intervention and mechanistic clinical evidence rather than epidemiological surveillance: although large surveillance programmes such as Childhood Obesity Surveillance Initiative (COSI) capture prevalence data across Central and Eastern Europe, longitudinal studies linking validated Mediterranean-diet adherence, eating-behaviour profiling and metabolic outcomes within active clinical pathways remain scarce in the region.

According to the WHO programme COSI, which has measured more than 1.3 million primary school children across 45 European countries, childhood overweight and obesity in long-term trends among children in Europe vary (6). From 2007 to 2017, prevalence decreased markedly in several Southern European countries–for example, overweight (including obesity) in Portuguese boys fell from 40.5 to 28.4%–while remaining stable or slightly increasing in Northern and Eastern European countries. In Lithuania, overweight in boys rose from 24.8 to 28.5% across the same surveillance period (1). By contrast, the global trend for BMI in children and adolescents has accelerated in parts of Asia and stabilised at high levels in many high-income countries, as documented in the NCD Risk Factor Collaboration’s pooled analysis of 128.9 million participants (4).

Lifestyle intervention-comprising dietary modification, increased physical activity and behavioural strategies–is the first-line treatment for childhood obesity in current clinical guidelines, with nutritional intervention as its central component (7). Among dietary patterns, the Mediterranean diet (MD) remains the most evidenced template. Its updated cultural and scientific representation places plant foods, whole grains, legumes, fish and extra-virgin olive oil at the core of a sustainable lifestyle pattern (8). Adherence to the MD is assessed in children and adolescents using the Mediterranean Diet Quality Index for children and youth (KIDMED) (9).

In addition to dietary patterns, behavioural traits modify weight trajectory in children. The Children’s Eating Behaviour Questionnaire (CEBQ) characterises eight appetitive traits (10), grouped into two domains: four food-approach subscales–Food Responsiveness (FR), Emotional Over-Eating (EOE), Enjoyment of Food (EF) and Desire to Drink (DD)- and four food-avoidance subscales–Satiety Responsiveness (SR), Slowness in Eating (SE), Emotional Under-eating (EUE) and Food Fussiness (FF).

These subscales are consistently associated with BMI Z-score in European paediatric populations (11, 12), and their joint use with KIDMED based diet quality assessment is recommended for personalised intervention design.

This narrative review summarises the European evidence base on nutritional intervention in obese children in the last 10 years and answers three main questions: (1) What evidence does European literature provide regarding Mediterranean Diet (MD) and the application of KIDMED based assessments in paediatric populations? (2) What are the characteristic outcomes and key predictors of effectiveness for multidisciplinary lifestyle interventions observed in recent European clinical trials? (3) How do European findings compare and contrast with results reported from other continents?

## 2 Methods

Given the broad scope of multidisciplinary strategies, eating behaviour constructs, and regional epidemiological patterns evaluated, a narrative review design was selected. This approach allowed for a comprehensive synthesis of clinical pathways, psychometric tools, and cross-cultural implementation barriers. To maintain methodological rigor, the search and reporting processes were structured in accordance with the Scale for the Assessment of Narrative Review Articles (SANRA) guidelines (13). We searched PubMed, Scopus and Web of Science for records published between January 2015 and 2026, combining the terms “childhood obesity” OR “paediatric obesity” with “Mediterranean diet,” “KIDMED,” “diet quality,” “eating behaviour” OR “Children’s Eating Behaviour Questionnaire”, “multidisciplinary” OR “lifestyle intervention”, and “non-alcoholic fatty liver disease” OR “MAFLD”; for the continental comparison these terms were additionally combined with “Europe,” “North America,” “Latin America,” “Asia,” “Middle East” and “Oceania.” Eligible sources were peer-reviewed studies published in English that concerned children and adolescents (approximately 2–18 years) and reported Mediterranean-diet adherence, eating-behaviour assessment, nutritional or multidisciplinary lifestyle intervention, or obesity prevalence and secular trends. Large surveillance and pooled analyses (e.g., COSI, HELENA, NHANES, NCD-RisC) were retained as reference points for prevalence and trends. Adult-only studies were excluded, with the exception of a small number of seminal instrument-development and mechanistic trials - for example the original KIDMED and CEBQ development studies and landmark Mediterranean-diet mechanistic trials - that were kept where foundational to the topic. This accounts for the inclusion of a limited number of references predating the 2015–2025 window. Consistent with the narrative design, no formal risk-of-bias scoring or quantitative meta-analysis was undertaken. Studies were selected and synthesised for their relevance to the three guiding questions. The key studies identified, with their design, instrument and main outcomes, are summarised in Table 1.

**Table 1:** Summary of key studies included in the review.

Study (author, year)	Region/Country	Design and population	Instrument/Approach	Main outcome
Serra-Majem et al., 2004 (9)	Spain	Instrument development; *n* = 3,850, 2–24 y (EnKid)	KIDMED development	KIDMED validated; only 46.4% high adherence
Kontogianni et al., 2008 (29)	Greece	Cross-sectional; representative children/adolescents	KIDMED	Low MD adherence nationally
Ashcroft et al., 2008 (10)	United Kingdom	Twin cohort; ages 4–10	CEBQ	Eating-behaviour traits stable over childhood
Grao-Cruces et al., 2013 (28)	Spain	Cross-sectional; *n* = 1,973, 11–18 y	KIDMED	30.9% optimal; rural/PA linked to adherence
Cakir et al., 2016 (34)	Turkey	Cross-sectional; obese children ± NAFLD	KIDMED vs. NAFLD	KIDMED ≤3: Se 66.9%, Sp 76.1% for NAFLD
Sánchez et al., 2016 (21)	Chile (GOCS)	Cohort; *n* = 1,058, 7–10 y	CEBQ	Same CEBQ–BMI gradients as Europe
Warschburger et al., 2016 (25)	Germany (EPOC)	RCT; *n* = 686	Multidisciplinary ± parent training	BMI-SDS − 0.24 at 12 m; no parent-training gain
Ojeda-Rodríguez et al., 2018 (20)	Spain	RCT; *n* = 107, abdominal obesity	MD-based lifestyle	↓ BMI-SDS, glucose, total cholesterol
Aparicio-Ugarriza et al., 2019 (19)	9 European countries (HELENA)	Cross-sectional; *n* = 2,330	KIDMED / MDS_A	Adherence linked to favourable biomarkers
Røed et al., 2021 (44)	Norway	RCT; *n* = 298 (Food4toddlers)	Parent-focused eHealth	↑ vegetable intake at follow-up 1
Ferrari et al., 2022 (46)	8 Latin American countries	Cross-sectional; *n* = 671 adolescents	Active transportation	Cycling ↓ overweight and central adiposity
Prieto-González et al., 2022 (42)	Saudi Arabia	Cross-sectional; *n* = 495 (college-aged)	KIDMED	MD adherence inversely correlated with BMI/fat
Lane et al., 2024 (43)	Australia (umbrella review)	Meta-analyses	UPF exposure	UPF ↑ obesity odds 1.55 (95% CI 1.36–1.77)
Chooi et al., 2024 (40)	Asia (TANGO)	RCT; *n* = 88	Asian-adapted MD	↓ liver fat 30%; weight −4.5%
Norman et al., 2024 (45)	Sweden	Cluster RCT; *n* = 353 families	School-based parental support	↑ healthy foods, ↓ sweet drinks, ↑ PA
Eggertsen et al., 2024 (23)	Denmark	RCT; *n* = 173	Multidisciplinary ± HIIT	BMI Z-score −0.11 (3 m), −0.20 (12 m)
Bober & Gaszyńska, 2025 (5)	Poland	Validation; *n* = 102, 10–18 y	KIDMED 2.0 PL	Strong inverse validity vs. BMI percentile
Imperiali et al., 2025 (30)	Italy	Community cohort; *n* = 1,331	Multidisciplinary	17.5% improved weight class; predictors identified
Votsi & Koutelidakis, 2025 (17)	Greece	Cross-sectional; *n* = 374 children	KIDMED + screen time	↑ screen time → ↓ MD adherence, ↑ fast food
Rangel-Quiñonez et al., 2025 (47)	Colombia	Experimental purchase study	Front-of-pack labelling	↓ purchase probability of ultra-processed foods

BMI, body mass index; CEBQ, Children’s Eating Behaviour Questionnaire; HIIT, high-intensity interval training; MD, Mediterranean diet; MDS_A, Mediterranean Diet Score for Adolescents; NAFLD, non-alcoholic fatty liver disease; PA, physical activity; RCT, randomised controlled trial; Se, sensitivity; Sp, specificity; UPF, ultra-processed food.

## 3 Nutritional intervention and its potential effects on childhood obesity

### 3.1 Mediterranean dietary pattern and KIDMED characteristics

The Mediterranean Diet (MD) is defined as a plant-forward dietary pattern characterized by the daily intake of fruits, vegetables, whole grains, legumes, nuts, and extra-virgin olive oil. This nutritional model includes moderate consumption of fish, dairy products, and poultry, while strictly limiting red and processed meat. Beyond its nutritional composition, the MD emphasizes cultural and lifestyle components, such as regular physical activity and adequate rest (8).

Recognized by the United Nations Educational, Scientific and Cultural Organization (UNESCO) as part of the Intangible Cultural Heritage of Humanity in 2010, the MD remains the most extensively evidenced dietary framework for cardiometabolic prevention throughout the lifespan (8, 14). As a key plant-forward dietary model, MD has been shown in meta-analyses to be more effective than conventional diets in managing body weight, blood pressure, and lipid profiles (15). A Mediterranean Lifestyle Pyramid specifically adapted for children and youth has recently been proposed as a practical tool for obesity prevention, integrating dietary recommendations with physical activity, adequate rest and conviviality within a sustainable framework (16).

KIDMED is a 16-item self-report instrument developed by Serra-Majem and colleagues in 2004 from data on 3,850 Spanish children and youth aged 2–24 years (the EnKid study), comprising twelve positively scored items reflecting Mediterranean aligned behaviours and four negatively scored items reflecting non-Mediterranean behaviours. Total score ranges from −4 to +12 and stratifies respondents into low (≤3), moderate (4–7) and high (≥8) adherence categories (9). KIDMED is a brief and effective screening tool designed for children and adolescents. As a brief self-report questionnaire, KIDMED can be completed directly by children and adolescents or administered to parents/guardians as proxy respondents, which is particularly useful in younger children (17). Its primary advantages include low respondent burden and a solid foundation in the Mediterranean dietary model, which is the gold standard for healthy eating (9). KIDMED has been adapted and psychometrically validated for several European paediatric populations. The Portuguese adaptation, validated in 185 adolescents from northern Portugal, demonstrated moderate two-week test–retest agreement and modest concurrent agreement with three-day dietary records (18). The Healthy Lifestyle in Europe by Nutrition in Adolescence (HELENA) study, conducted across nine European countries with n = 2,330 adolescents aged 12.5–17.5 years, showed that both the continuous KIDMED score and the alternative Mediterranean Diet Score for Adolescents (MDS_A) were positively associated with serum 25-hydroxyvitamin D, vitamin C, plasma folate, holo-transcobalamin, *β*-carotene and n-3 fatty acid concentrations, and inversely associated with serum trans-fatty acid levels (19). The Polish adaptation, KIDMED 2.0 PL, was validated in 102 children and adolescents aged 10–18 years. It showed good two-week test–retest reliability (Spearman’s *ρ* = 0.876) and strong inverse criterion validity against BMI percentile (*ρ* = −0.854), with item-level analysis revealing relatively common fruit and vegetable intake but low intake of legumes (11.8% ≥ 3×/week), extra-virgin olive oil at home (18.6%), and high consumption of processed sweets (74.5% ≥ 1×/week) and sugar-sweetened beverages (60.8% ≥ 1×/week) (5).

### 3.2 Nutritional intervention components and potential effects on childhood obesity

Current European protocols for treating childhood obesity use a multidisciplinary approach. These interventions combine different, well-matched strategies that are applied at the same time. Typically, they integrate nutritional education with lifestyle changes to ensure long-term health benefits.

#### 3.2.1 Dietary pattern (MD, DASH, plant forward diet)

MD based and other plant-forward dietary patterns improve cardiometabolic markers even when delivered without explicit caloric restriction. In the Spanish randomized controlled trial by Ojeda-Rodríguez et al., an MD-based intervention in 107 children with abdominal obesity produced significant reductions in BMI Z-score and in plasma glucose and total cholesterol, with concurrent improvements in nutrient adequacy for calcium, iodine and vitamin D (20). The DASH (Dietary Approaches to Stop Hypertension) pattern emerged as a top-three weight-loss diet alongside Atkins and Zone in the BMJ 2020 network meta-analysis of 14 named diets in adults (15). Paediatric DASH evidence is more limited. The broader green-Mediterranean variant (amplified with Mankai green plant protein, walnuts and green tea) reduced intrahepatic fat by 38.9% in adults with abdominal obesity in the Israeli DIRECT-PLUS trial (14), indirectly informing paediatric NAFLD strategy.

#### 3.2.2 Eating behaviour modification (CEBQ)

Across European cohorts, food-approach subscales (FR, EOE, EF, DD) are positively associated with BMI Z-score, while food-avoidance subscales (SR, SE, FF) are negatively associated (11, 12, 21). In a Bosnian cross-sectional sample of 2,500 children aged 3–10 years, BMI Z-score increased linearly with food-approach scores and decreased linearly with food-avoidance scores (12). Equivalent associations were reported in Spanish, Chilean and Saudi populations (11, 21). Eating behaviour traits show strong continuity from age 4 to age 10 in twin-design studies, with test–retest correlations of 0.44–0.55 across most subscales-comparable to stable personality traits (10). These findings support routine CEBQ based screening alongside diet quality screening in paediatric obesity treatment intake.

#### 3.2.3 Multidisciplinary lifestyle interventions

The European multidisciplinary core typically comprises a paediatric obesity doctor, dietitian, psychologist, physiotherapist and diabetologist, integrated into a structured care pathway (22–25). Additional components - high-intensity interval training (HIIT), parent training, family-based behavioural therapy - have shown benefits in recent European RCTs (23, 25). The current European evidence supports prioritising the multidisciplinary core over single component intervention. Whether this specialist configuration is required, or whether the same functions can be delivered through other professional profiles, is considered in the continental comparison (Section 5.6).

#### 3.2.4 Educational and family components

Free to attend nutrition and lifestyle education workshops for patients and parents/guardians are integrated into many European multidisciplinary obesity treatment infrastructures. School-based food-environment policies - direct provision of fruits and vegetables, competitive food/beverage standards, and school meal standards - improved targeted dietary behaviours in a meta-analysis of 91 interventions, although effects on adiposity were inconsistent (26). School-based gardening, cooking and nutrition interventions in the United States increased vegetable intake but did not reduce BMI in the Texas Sprouts cluster RCT of 3,135 children (27), suggesting that single environment exposures are insufficient and should be combined with clinical pathways for children with established obesity.

## 4 Nutritional intervention and its influence on childhood obesity outcomes - current state of knowledge

### 4.1 MD adherence in European paediatric populations

Low MD adherence in European children and adolescents is consistent across countries and decades. In the original Spanish EnKid sample, 4.2% of participants showed very low KIDMED scores and only 46.4% achieved high scores, with marked geographical and socioeconomic variation within Spain (9). Spanish data from rural and urban adolescents in the south showed that 30.9% of 1,973 11–18 year olds reported optimal diet quality, with rural settings, younger age, greater life satisfaction and higher physical activity engagement all associated with better adherence (28). A comparable pattern of low adherence has been reported in a representative sample of Greek children and adolescents, where relatively few participants achieved optimal KIDMED scores (29). The HELENA evidence reaffirms these patterns at the cross-European level (19). The Polish KIDMED 2.0 PL validation reproduces the pattern in a Central-European setting and identifies specific item level gaps in legumes, extra-virgin olive oil and whole grains (5).

### 4.2 Multidisciplinary lifestyle interventions-European evidence

Recent European RCTs and large observational cohorts report consistent reductions in BMI Z-score under multidisciplinary intervention. The (23) Danish RCT randomised 173 children with obesity to a 12-month multidisciplinary lifestyle intervention with or without a 12-week HIIT supplement. BMI Z-score fell by 0.11 at 3 months and by 0.20 at 12 months (23). The German EPOC RCT (n = 686) reported a within-group BMI-SDS reduction of 0.24 at 12 months, and no incremental benefit from cognitive-behavioural parent training (25). The Italian Emilia-Romagna community programme (*n* = 1,331) reported that 17.5% of children improved their weight class and 32.5% achieved a Δ-BMI greater than one unit at follow-up (30). The Dutch Kids4Fit cohort study from deprived areas in Rotterdam (*n* = 154) showed a non-significant trend toward lower BMI Z-score and a statistically significant reduction in waist circumference (31).

Adjuncts to the multidisciplinary core have shown mixed effects. The Norwegian FBSFT (24) and Spanish PREDIKID (22) family-based protocols are now in long-term follow-up and represent the next wave of European evidence on family centred approaches in paediatric obesity. Predictors of clinical response are consistent across the European literature and include lower baseline BMI severity (paradoxically associated with higher response rate, partly through regression to the mean), older age, breakfast adherence, and the absence of obese parents (30). The Italian cohort reported 1.41 fold higher adjusted odds of weight class improvement among children older than 8 years compared to younger children, and a halving of odds among those with at least one obese parent (30).

MD based interventions in European obese children are less numerous but converge with the broader literature. The Spanish Ojeda-Rodríguez 2018 RCT is the most directly relevant trial. The Spanish Basque study by Jauregibeitia et al. (32) adds biomarker depth by combining KIDMED scoring with mature red blood cell membrane fatty-acid profiling in 209 children, demonstrating a pro-inflammatory red cell membrane signature in children with obesity that tracks with low MD adherence.

### 4.3 Paediatric NAFLD

Paediatric non-alcoholic fatty liver disease (NAFLD), increasingly designated metabolic dysfunction-associated fatty liver disease (MAFLD/MASLD) is the most frequent chronic liver condition in children with obesity and represents a critical downstream target for nutritional intervention (33). In a Turkish cross-sectional study, KIDMED scores were significantly lower in obese children with NAFLD than in obese children without NAFLD (2.6 ± 2.4 vs. 4.6 ± 1.2; *p* < 0.05). KIDMED score ≤ 3 had a sensitivity of 66.9% and a specificity of 76.1% for predicting NAFLD in obese children (34). The narrative review of Rupasinghe et al. (33) summarises that 7–10% weight loss reverses MAFLD in most paediatric patients and that the MD shows promise as a reversal supporting dietary template. The Mexican systematic review and meta-analysis of 31 paediatric MASLD trials confirmed significant improvements in alanine aminotransferase, aspartate aminotransferase, BMI and the homeostatic model assessment of insulin resistance (HOMA-IR) under combined supplement and lifestyle intervention (35).

## 5 Continental comparison: Europe versus other continents

Global variations in childhood obesity and its dietary drivers are primarily observed in three areas: the overall prevalence of the condition, the increase over time, and the extent to which the MD is culturally embraced as a viable intervention model.

### 5.1 North America

US paediatric obesity prevalence is among the highest in high-income countries. The National Health and Nutrition Examination Survey (NHANES) analysis by Skinner et al. (36) reported a positive linear trend across all definitions of overweight and obesity between 1999 and 2016 in 2–19 year olds, with no evidence of decline at any age. The 20-year analysis by Tsoi et al. (37), covering 35,907 NHANES participants between 1999 and 2018, reported that obesity prevalence rose from 14.7 to 19.2% and severe obesity from 3.9 to 6.1% across the two decade window, with a markedly higher prevalence (24.4%) among children from Spanish-speaking US households compared with English-speaking households. By contrast with European COSI countries, the absolute prevalence in the US is roughly double that of high performing Southern European countries - Portugal and Greece - in primary school children of comparable age (1, 36).

US trial evidence on lifestyle intervention is heterogeneous. The Texas Sprouts school-based gardening, cooking and nutrition cluster RCT (*n* = 3,135 children) increased vegetable intake by 0.48 servings/day but did not reduce BMI (27). Family-based behavioural treatment frameworks - adapted from the Family-based behavioural social facilitation treatment (FBSFT) - show promise. Their European counterparts are now in long-term follow-up (24). The absence of a paediatric MD adapted RCT directly comparable to the European Spanish evidence remains a notable gap in the US literature.

### 5.2 Latin America

Latin America presents a complex food-environment landscape characterised by rapid nutrition transition, ultra processed food market penetration, and strong policy driven counter-responses (ex. Chile’s front of pack warning labels, Mexico’s sugar sweetened beverage tax) (38). The Duran et al. (38) review synthesises the Latin American food environment evidence base and identifies an integrated set of policy levers as the most promising route to childhood obesity prevention in the region. The Chilean GOCS cohort (*n* = 1,058 children aged 7–10) shows the same CEBQ subscale BMI Z-score gradients reported in European cohorts: BMI Z-score increases with food-approach scores and decreases with food-avoidance scores (21), supporting the cross-cultural applicability of the CEBQ instrument. The Mexican-led MASLD/obesity meta-analysis (35) adds the most recent paediatric-NAFLD evidence and highlights the need for high-quality lifestyle and dietary trials in diverse Latin American populations.

### 5.3 Asia

Asian paediatric obesity prevalence has been rising at a rate that exceeds the European pattern. Hong et al. (39) summarised the Chinese trends: prevalence has increased for decades with marked geographical variation, the highest absolute prevalence persists in Northern China, and obesity related metabolic disease is becoming more frequent even where overall prevalence has stabilised. The NCD Risk Factor Collaboration’s pooled analysis of 128.9 million participants documented that the rise in BMI has accelerated in east and south Asia for both sexes and in south-east Asia for boys, in contrast to the plateau observed in north-western Europe and the high-income English-speaking and Asia-Pacific regions (4). The (40) TANGO RCT (*n* = 88 Chinese females with NAFLD) demonstrated that an Asian-adapted MD reduced liver proton density fat fraction by 30%, body weight by 4.5%, and triglycerides - providing the strongest paediatric/young adult evidence to date that culturally adapted MD based intervention is effective in non-Mediterranean Asian populations. Direct paediatric MD trials in Asia remain rare and constitute a research priority.

### 5.4 Middle East

Middle East paediatric obesity prevalence is among the highest globally. Gulf states report >20% paediatric obesity in cross-sectional studies (4, 41). Cultural and dietary proximity to the broader Mediterranean basin makes the Middle East a natural setting for KIDMED-based research. Prieto-González et al. (42) reported that, among 495 college-aged Saudi males, MD adherence assessed by KIDMED was strongly inversely correlated with BMI (*r* = −0.61), body fat percentage (*r* = −0.56) and abdominal girth (*r* = −0.56), and moderately inversely correlated with fasting glucose. The Israeli DIRECT-PLUS green-Mediterranean trial in adults (14), although not paediatric, established a regional evidence anchor for MD-based intervention. The EAS-FH cardiovascular registry of 35,540 patients with familial hypercholesterolaemia across 50 countries reported the highest prevalence of paediatric overweight and obesity within Northern Africa and Western Asia, contributing to elevated atherosclerotic-cardiovascular-disease risk independent of low-density lipoprotein (LDL) cholesterol (41).

### 5.5 Australia and Oceania

Australia and New Zealand follow the high-income, English-speaking trajectory characterized by a stabilized yet elevated prevalence of paediatric obesity (4). Polynesia and Micronesia experienced the highest age-standardised BMI increase in the NCD-RisC 1975–2016 analysis among children. Obesity prevalence in 2016 was above 30% in girls in Nauru, the Cook Islands and Palau, and in boys in the Cook Islands, Nauru, Palau, Niue and American Samoa (4). Australian-led research has contributed major ultra processed food (UPF) evidence. The umbrella review by Lane et al. (43) of epidemiological meta-analyses linked greater UPF exposure to a 1.55-fold higher odds of obesity (95% CI 1.36–1.77) in addition to elevated risks of cardiovascular mortality, type 2 diabetes, depressive outcomes and adverse sleep. UPF intake is also a key concern across European children but is more comprehensively studied in the United Kingdom, Australia and Brazil to date.

### 5.6 Intervention strategies across settings: shared determinants

Beyond prevalence, the determinants and delivery of intervention converge across continents. Parental and caregiver behaviour is a consistent lever. Parent-focused digital interventions that reshape the early food environment have improved toddlers’ vegetable intake in Norway (44), and parental adherence to the Mediterranean diet tracks closely with children’s own adherence and weight status (17). These findings support administering KIDMED to parents/guardians as proxy respondents for younger children and targeting the family food environment rather than the child in isolation.

Family- and school-based programmes show comparable benefits internationally. The Swedish Healthy School Start Plus cluster-randomised trial delivered parental support through the school setting and improved children’s intake of healthy foods, reduced sweet-beverage intake and increased physical activity in disadvantaged areas (45), echoing the European family-based protocols (22, 24) and United States family-based behavioural treatments. Because eating-behaviour traits and food preferences are established early and are stable over childhood (10), interventions that shape preferences in early childhood are likely to have the most durable effects.

School food environments and active-transport infrastructure complement clinical pathways. School food-environment policies improved targeted dietary behaviours across 91 interventions, although effects on adiposity were inconsistent (26), and in Latin America cycling for active transportation was associated with lower odds of overweight and central adiposity across eight countries (46).

Food marketing and screen exposure are shared risk determinants. Greater screen time is associated with poorer Mediterranean-diet adherence and higher fast-food and soft-drink consumption in schoolchildren (17). Population-level countermeasures include front-of-pack warning labelling, which reduced the purchase probability of processed and ultra-processed foods in Colombia (47), mirroring Chile’s front-of-pack scheme and Mexico’s sugar-sweetened-beverage tax (38), and national programmes such as Brazil’s PROTEJA strategy for childhood-obesity prevention in cities (48).

Finally, the professional configuration through which these functions are delivered varies globally. The specialist European core described above-paediatric obesity physician, dietitian, psychologist, physiotherapist and diabetologist (Section 3.2.3)-is frequently unavailable in lower-resource and non-European settings, where task-sharing with primary-care clinicians, school nurses, community health workers and families is more common (45). Current guidance frames comprehensive, intensive health-behaviour and lifestyle treatment as the standard of care irrespective of the specific roster (7). The available evidence suggests that outcomes depend on the multidisciplinary functions delivered-dietary, behavioural, physical-activity and family support-rather than on a fixed professional profile, which has direct implications for scalability across diverse health systems.

### 5.7 Patterns across the world

Three patterns emerge from continental comparisons. First, dietary-quality predictors of paediatric obesity-low intake of legumes, whole grains, extra-virgin olive oil, high intake of processed sweets and sugar-sweetened beverages-are broadly consistent between European, Latin American, Middle East and Asian samples, supporting the cross-cultural applicability of the KIDMED-based screening template (5, 9, 18, 28, 42). Second, the magnitude of BMI Z-score reduction achievable in multidisciplinary intervention is broadly comparable across continents (0.10–0.25 Z-units in 3–12 months) but the absolute baseline prevalence differs, with the United States, parts of Latin America and Polynesia at the high end and high-performing Southern European countries at the lower end (1, 4, 36). Third, secular trends are heterogeneous: stabilisation in northern, western and southern Europe and high-income Asia-Pacific contrasts with continued acceleration in east and south Asia and persistent high prevalence in the Middle East and the United States (1, 4, 36, 39). These patterns argue for a culturally adaptive but instrument-harmonised approach, in which the KIDMED template is locally adapted and combined with CEBQ-based behavioural screening, with intervention components tailored to the local food environment and policy infrastructure.

The potential for cross-regional transferability of the Mediterranean diet should not be overestimated. The current evidence base relies predominantly on studies conducted within Mediterranean populations, where agricultural systems, high food availability, and cultural familiarity facilitate adherence. Outside Southern Europe, structural and economic barriers emerge: extra-virgin olive oil remains comparatively expensive, fresh produce and legumes are less accessible, and traditional dietary patterns in Central and Eastern Europe, as well as in many low- and middle-income regions, do not naturally center on these core components. Consequently, successful cross-cultural application requires adapting Mediterranean dietary principles to locally available, affordable, and culturally familiar substitutes, rather than attempting direct, unadapted implementation (16, 29).

## 6 Future perspective

The literature review identifies three main research gaps. First, longitudinal Central- and Eastern-European data linking validated MD adherence measurement, eating-behaviour profiling, and metabolic outcomes within an active clinical pathway are scarce. Second, the relative contribution of regression-to-the-mean versus genuine treatment resistance to the lower response rate observed in severely obese subgroups remains unresolved in the European literature (23, 30). Specifically, among children with severe obesity, the mean reduction in BMI Z-score is consistently lower. However, it remains unclear to what extent this finding reflects true treatment resistance rather than a statistical artifact. Because individuals with lower baseline severity are more susceptible to regression to the mean-where extreme measurements naturally shift toward the average upon follow-up-their observed response may be inflated, thereby artificially widening the apparent gap between severity groups. Addressing this methodological challenge would benefit from time-stratified analyses and multivariable adjustment for baseline severity in subsequent trials. Third, formal assessment of the integrated KIDMED + CEBQ + metabolic biomarker approach as a single predictive model has not been published in any European paediatric cohort with paired longitudinal data, although emerging Asian work points to its feasibility (40).

These gaps are global rather than uniquely European. Current evidence strongly supports population-level policy measures- including front-of-pack warning labels (47), sugar-sweetened beverage taxation, and national initiatives like Brazil’s PROTEJA- alongside family- and school-based interventions (44, 45). However, direct paediatric Mediterranean diet trials outside the Mediterranean basin remain limited. Furthermore, the real-world feasibility of Mediterranean dietary patterns in regions where extra-virgin olive oil, legumes, and fresh produce are economically restrictive or culturally unfamiliar is under-researched (16, 29). Consequently, future research should integrate standardized screening instruments with culturally adapted, economically viable dietary targets, while evaluating multi-component intervention packages that incorporate countermeasures against screen time and food marketing (17, 46).

A practical screening pathway follows from this evidence and is summarised as a concept flowchart (Figure 1): (1) a child with overweight or obesity enters a multidisciplinary pathway; (2) diet quality (KIDMED) and eating behaviour (CEBQ) are screened in parallel, by self- or proxy-report; (3) results are stratified (low/moderate/high MD adherence; food-approach versus food-avoidance profile); (4) a personalised intervention is matched to the profile and to locally feasible dietary targets; and (5) metabolic markers (BMI Z-score, ALT/GGTP, the TG/HDL ratio) are monitored, with re-screening at predefined follow-up points.

**Figure 1:**
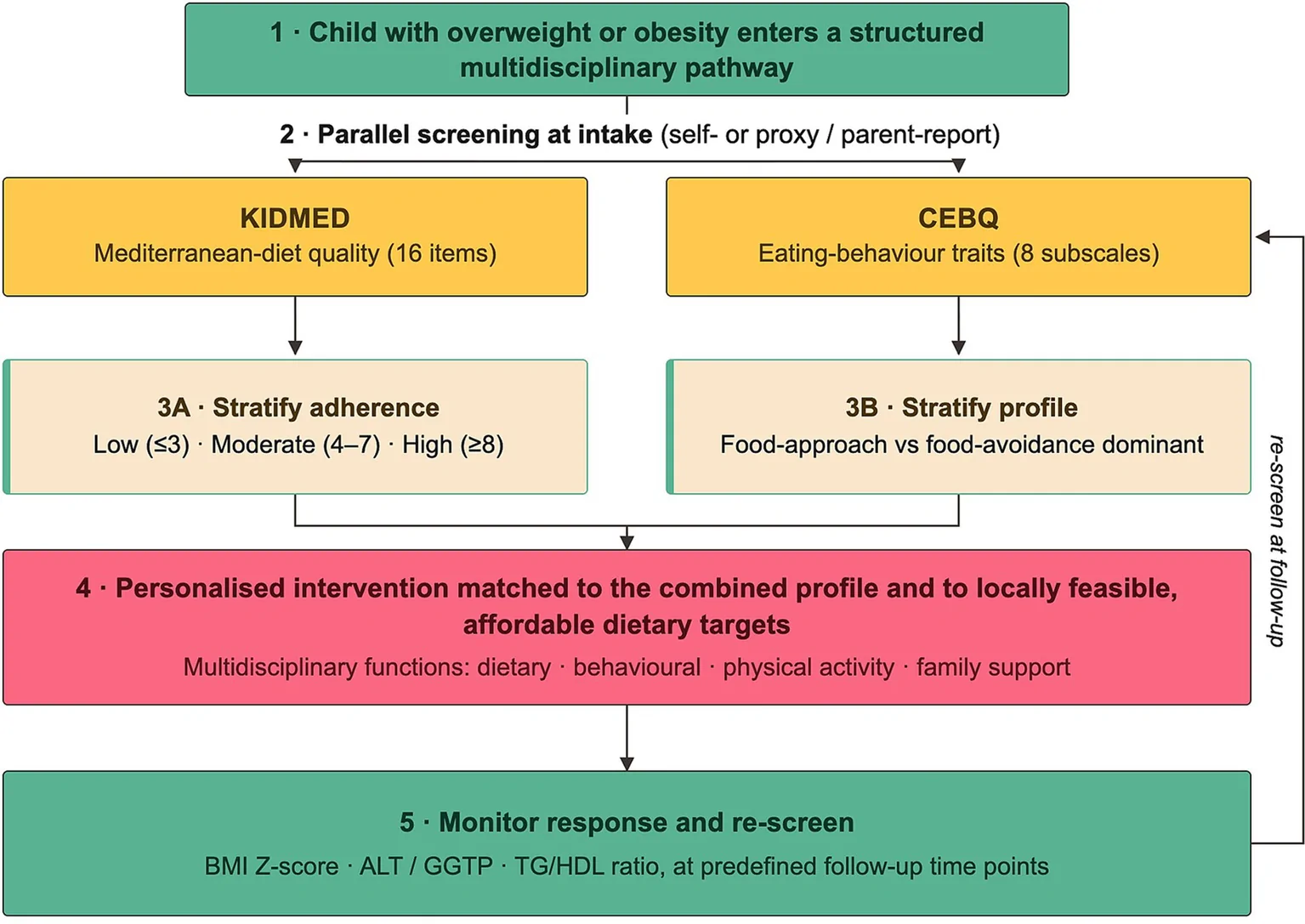
Integrated KIDMED + CEBQ paediatric clinical care pathway. A child with overweight or obesity entering a structured multidisciplinary pathway is screened in parallel for Mediterranean-diet quality (KIDMED) and eating behaviour (CEBQ), by self- or proxy/parent-report. KIDMED adherence is stratified as low (≤3), moderate (4–7) or high (≥8) and the CEBQ profile as food-approach- or food-avoidance-dominant; a personalised intervention is then matched to the combined profile and to locally feasible, affordable dietary targets, delivering the core multidisciplinary functions (dietary, behavioural, physical-activity and family support). Treatment response is monitored (BMI Z-score; alanine aminotransferase/gamma-glutamyl transpeptidase; triglyceride-to-HDL-cholesterol ratio) with re-screening at predefined follow-up points. ALT, alanine aminotransferase; BMI, body mass index; CEBQ, Children’s Eating Behaviour Questionnaire; GGTP, gamma-glutamyl transpeptidase; KIDMED, Mediterranean Diet Quality Index for children and youth; TG/HDL, triglyceride-to-HDL-cholesterol ratio.

Four implications for clinical practice follow:

KIDMED-based MD adherence screening should be integrated as a routine, low-cost element of paediatric obesity-treatment intake, using the country-specific validated adaptation where available (5, 18).

CEBQ-based behavioural screening should be performed alongside diet-quality screening to inform personalized intervention design (11, 12).

The multidisciplinary core should be prioritised in European obesity-treatment infrastructure investment over single-component adjuncts, given the consistent lack of incremental BMI Z-score benefit from single-component adjunct trials (23, 25).

Hepatic-load markers (ALT, gamma-glutamyl transpeptidase, GGTP) and the validated paediatric triglyceride-to-HDL-cholesterol (TG/HDL) should be retained in the routine monitoring panel as sensitive markers of metabolic improvement under MD based intervention (33–35).

## 7 Conclusion

Over the past decade, European research has established the Mediterranean diet as a premier framework for paediatric obesity prevention and treatment. Standardized screening via KIDMED and the CEBQ provides a validated, implementation-ready strategy to identify dietary gaps and appetitive traits within clinical settings. Across global cohorts, structured multidisciplinary interventions consistently demonstrate reductions in BMI Z-scores, with response efficacy largely driven by early intervention, lower baseline severity, regular breakfast consumption, and active family involvement. Because structural barriers to Mediterranean diet adherence exist outside Southern Europe, successful cross-cultural translation depends on adapting dietary targets to local food availability, costs, and culinary traditions.

Current evidence highlights three fundamental dimensions of childhood obesity management: (1) effective interventions rely on structured, multidisciplinary lifestyle programs that integrate dietary modification, physical activity, behavioural counseling, and active family engagement, backed by supportive population-level policies; (2) while these strategies demonstrate cross-regional applicability when tailored to local food environments, their implementation yields the greatest potential impact in high-burden regions provided component foods remain affordable and culturally aligned; and (3) intervention efficacy is consistently highest among younger children, individuals with lower baseline obesity severity, those with non-obese parents, and children who adhere to regular breakfast consumption. Consequently, routine integration of harmonized KIDMED and CEBQ tools into structured multidisciplinary care represents an actionable priority, particularly for underserved paediatric populations in Central and Eastern Europe.
